# Fine Tuning of ROS, Redox and Energy Regulatory Systems Associated with the Functions of Chloroplasts and Mitochondria in Plants under Heat Stress

**DOI:** 10.3390/ijms24021356

**Published:** 2023-01-10

**Authors:** Nobuhiro Suzuki

**Affiliations:** Department of Materials and Life Sciences, Faculty of Science and Technology, Sophia University, 7-1 Kioi-cho, Chiyoda, Tokyo 102-8554, Japan; n-suzuki-cs6@sophia.ac.jp; Tel.: +81-3-3238-3884

**Keywords:** chloroplast, energy, heat stress, mitochondria, reactive oxygen species (ROS)

## Abstract

Heat stress severely affects plant growth and crop production. It is therefore urgent to uncover the mechanisms underlying heat stress responses of plants and establish the strategies to enhance heat tolerance of crops. The chloroplasts and mitochondria are known to be highly sensitive to heat stress. Heat stress negatively impacts on the electron transport chains, leading to increased production of reactive oxygen species (ROS) that can cause damages on the chloroplasts and mitochondria. Disruptions of photosynthetic and respiratory metabolisms under heat stress also trigger increase in ROS and alterations in redox status in the chloroplasts and mitochondria. However, ROS and altered redox status in these organelles also activate important mechanisms that maintain functions of these organelles under heat stress, which include HSP-dependent pathways, ROS scavenging systems and retrograde signaling. To discuss heat responses associated with energy regulating organelles, we should not neglect the energy regulatory hub involving TARGET OF RAPAMYCIN (TOR) and SNF-RELATED PROTEIN KINASE 1 (SnRK1). Although roles of TOR and SnRK1 in the regulation of heat responses are still unknown, contributions of these proteins to the regulation of the functions of energy producing organelles implicate the possible involvement of this energy regulatory hub in heat acclimation of plants.

## 1. Introduction

Heat stress caused by unusual temperature increase severely affects plant growth and crop production. It has been also estimated that global temperature will increase 2.0–4.9 °C by the end of 21st century [1]. It is therefore urgent to elucidate the mechanisms underlying responses of plants to heat stress and establish the strategies to enhance heat tolerance of crops.

Heat stress can cause detrimental effects on growth and development via disturbing various physiological traits in plants and crops [2]. Especially, the chloroplasts and mitochondria as well as the plasma membrane are known to be highly sensitive to heat stress [3,4]. Many studies reported the negative impacts of heat stress on activities of the key enzymes involved in the metabolisms associated with photosynthesis and respiration [3,4]. It was also demonstrated that disruptions of the electron transport chains in the chloroplasts and mitochondria resulted in the excess accumulation of reactive oxygen species (ROS) such as ^1^O_2_, O_2_^−^ and H_2_O_2_ which can cause damages on cells under heat stress [3,4]. Despite their toxic potential, however, ROS are also known to play important roles as signaling molecules required for acclimatory responses of plants to heat stress [5,6,7]. ROS accumulating in different cellular compartments at various concentrations might be required for the creation of specific signals suitable for acclimation to different types of stresses [8,9]. A recent study demonstrated that ROS accumulation increased in the cytosol and nucleus in response to heat stress [10]. Such a pattern of ROS accumulation in cells might be associated with reprograming of the transcriptome to tailor heat stress responses [10]. ROS required for signaling can be also produced via NADPH oxidases (RESPIRATORY BURST OXIDASE HOMOLOGUEs; RBOHs) localized in the plasma membrane [11,12,13]. ROS produced by RBOHs might activate various heat acclimatory pathways involving several heat response transcription factors [5]. For example, ROS affect the translocation of redox regulated transcription factors such as HEAT SHOCK TRANSCRIPTION FACTORs (HSFs) and MULTIPROTEIN BRIDGING FACTOR 1c (MBF1c) which are known to be heat responsive to the nucleus [14,15]. A previous study demonstrated that HSFA2 might regulate a key regulator of unfolded protein response in the endoplasmic reticulum (ER-UPR) via modulation of ROS signaling during heat stress [16]. In addition, ROS generated in the chloroplasts and mitochondria are known to activate several stress acclimatory mechanisms via retrograde signaling, the regulatory systems of nuclear gene expression [17,18]. These findings suggest that the balance of ROS level among different organelles including the chloroplasts and mitochondria needs to be strictly controlled via producing and scavenging systems under heat stress.

Previous studies uncovered several mechanisms that protect the chloroplasts and mitochondria against heat stress. HEAT SHOCK PROTEINs (HSPs) were shown to be involved in the maintenance of various biological processes including regulation of ROS level and electron transport in the chloroplasts and mitochondria [19,20,21]. Indeed, these organelles are well known as major sources of ROS production [22,23]. It might be therefore reasonable to speculate that the mechanisms to prevent excess ROS accumulation in the chloroplasts and mitochondria have been evolved in plant cells. Retrograde signaling might also play key roles to maintain functions of these organelles under heat stress via activation of pathways involving HSPs [24,25]. ALTERNATIVE OXIDASE (AOX), a marker of the retrograde signaling activity which can prevent over-reduction of the electron transport chain was also implicated in heat responses in the mitochondria [26,27].

As regulation of some of these pathways are energy-consuming processes, heat response in plant cells might rely on the balance of the activities between the chloroplasts and mitochondria [4,28]. Heat stress negatively affects photosynthesis, leading to depletions of sugars and starches. Depletions of these substrates for the respiratory metabolisms impacts on the efficiency of energy production which is required for the activity of heat response pathways [4,29]. Thus, control of respiration/photosynthesis ratio by improving rate of the photosynthetic CO_2_ fixation and reducing energy costs might be important to enhance the ability of plants to acclimate to heat stress [29].

TARGET OF RAPAMYCIN (TOR) and SNF1-RELATED PROTEIN KINASE 1 (SnRK1) are known to play key roles to switch signals underlying growth, development and stress responses depending on energy status [30,31,32,33]. Under the conditions in which plants can effectively produce energy via photosynthesis and respiration, TOR activates energy consuming mechanisms to boost growth and development [31]. In contrast, under the stressed conditions that can cause energy depletion, SnRK1 inhibits the energy-consuming growth mechanisms regulated by TOR while activating stress responses as well as mechanisms to obtain energy from alternative sources [30,32]. TOR and SnRK1 were shown to target many proteins involved in a broad range of biological processes including the maintenance of metabolisms associated with photosynthesis and respiration as well as retrograde signaling [30,31,32,34,35]. However, integrations of TOR- and SnRK1-dependent mechanisms with functions of the chloroplasts and mitochondria are still not understood and the roles of TOR and SnRK1 in the regulation of heat stress responses need to be elucidated.

In this review, the author will discuss effects of heat stress on the functions of the chloroplasts and mitochondria and the mechanisms underlying acclimatory responses of these energy regulating organelles to heat stress, including retrograde signaling. The author will also suggest possible integration of mechanisms underlying heat stress responses of the chloroplasts and mitochondria with the energy regulatory system involving TOR and SnRK1.

## 2. Effects of Heat Stress on the Chloroplasts and Mechanisms to Counteract Them

The chloroplast is well known as one of the most sensitive organelles to heat stress [36]. It has been reported that heat stress caused enlargement of the chloroplasts as well as increase in the number of plastid vesicles [37]. Such alterations might occur due to the disruption of the thylakoid membrane caused by the changes in redox status of the chloroplasts [3,29]. Photosystem II (PSII), which is embedded in the thylakoid membrane, is also known as a sensitive target of heat stress in the chloroplasts [36]. Heat stress can perturb PSII function via increasing fluidity of the thylakoid membranes, leading to falling off of the PSII light-harvesting complex from the thylakoid membrane, as well as dissociation of the oxygen evolving complexes [38,39,40,41,42]. These effects on PSII induced by heat stress result in inhibition of photosynthetic electron transport in PSII and decrease in ATP synthesis, leading to acceleration of ROS production [17].

Under unfavorable conditions, ROS including superoxide anion (•O_2_^−^), hydrogen peroxide (H_2_O_2_), hydroxyl radical (•OH) and singlet oxygen (^1^O_2_) can be generated mainly in the reaction centers of PSI and PSII in the thylakoid membrane [18,22]. ^1^O_2_, which is produced as a byproduct of photosynthesis via excitation of ground-state triplet oxygen [22,36], can severely damage D1 and D2 proteins, the reaction center proteins in PSII [43]. Particularly, D1 protein is susceptible to ^1^O_2_ due to its localization to the proximity of ^1^O_2_ production site [44]. It has been also reported that a large amount of chlorophyllide-a, a precursor of chlorophyll synthesis, dramatically increased ^1^O_2_ production in *Arabidopsis* mutant deficient in CHLOROPHYLL SYNTHASE (CHLG) under heat stress [45]. This finding suggests a key role of CHLG in the maintenance of cellular ROS level [45]. Significance of the maintenance of chlorophyll synthesis in heat tolerance can be further supported by the finding that mutants with delayed senescence phenotypes showed enhanced tolerance to heat stress in various plant species [46,47]. Disruptions of the mechanisms responsible for the regulation of H_2_O_2_ and O_2_^-^ also cause negative impacts on plant cells [48,49]. Downregulation of thylakoid ASCORBATE PEROXIDASE (APX) or Cu/Zn SUPEROXIDE DISMUTASE (Cu/ZnSOD), enzymes responsible for scavenging H_2_O_2_ or O_2_^−^, respectively, in the chloroplasts resulted in retardation of growth [48]. In addition, heat stress triggered degradation of Cu/ZnSOD, while inducing activity of CATALASE (CAT) [50], suggesting that heat stress can alter the balance between H_2_O_2_ and O_2_^−^. Alterations in the activities of antioxidant enzymes and the balance of ROS production can be critical for the function of PSII under heat stress [17].

Heat sensitivity of the Calvin–Benson cycle has been also reported in previous studies [17,51]. In this cycle, activity of RIBULOSE-1,5-BISPHOSPHATE CARBOXYLASE/OXYGENASE (RUBISCO), an enzyme responsible for the fixation of CO_2_ is known to be inhibited by heat stress [17]. Inhibition of RUBISCO activity occurs due to inhibition of another enzyme, RUBISCO ACTIVASE (RCA) that uses the energy from ATP hydrolysis to restore RUBISCO [17]. Thus, inhibition of electron flow leading to decrease in ATP synthesis in the thylakoid membrane might be one of the major limiting factors of the Calvin–Benson cycle during heat stress. Affinity of RUBISCO for CO_2_ also decreases under heat stress, which tends to accelerate the oxygenase reaction of this enzyme. The process enhanced by the oxygenase activity of RUBISCO is known as photorespiration, which reduces efficiency of photosynthesis [51]. Stability of a chaperone required for the maintenance of the RUBISCO structure might be a key for the activity of CO_2_ fixation in the stroma. A recent study demonstrated that DnaJ protein in tomato (SlCDJ2) plays important roles to maintain the activity of CO_2_ fixation via RUBISCO under heat stress [52]. In addition, several other enzymes involved in the Calvin–Benson cycle were shown to be regulated by the thioredoxin (TRX)-dependent mechanisms that perceive electrons derived from the electron transport chain in the thylakoid membrane [22]. Thus, inhibition of electron transport caused by heat stress might negatively affect activities of these enzymes.

Plants possess mechanisms to attenuate damages on the chloroplasts caused by heat stress. Heat priming, pre-exposure to mild heat treatment that enhances heat tolerance of plants, contributes to the maintenance of redox regulation by activating ROS scavenging enzymes such as SUPEROXIDE DISMUTASE (SOD) and GLUTATHIONE REDUCTASE (GR) in the chloroplasts as well as mitochondria [53]. THIOREDOXIN-LIKE 1 (TRXL1) in the chloroplasts also activates NADP-DEPENDENT MALATE DEHYDROGENASE and attenuates production of O_2_^−^, leading to enhancement of heat tolerance as well as disease resistance [54]. SEROTONIN N-ACETYLTRANSFERASE in tomato (SlSNAT), an enzyme required for melatonin synthesis was shown to be involved in ROS scavenging [55]. SlSNAT localized in the chloroplasts interacts with HSP40 [55]. Overexpression of SlSNAT resulted in enhanced heat tolerance of the transgenic plants accompanied by enhanced melatonin accumulation, higher capacity of ROS scavenging and upregulation of heat response transcripts. Significance of melatonin in heat acclimation has been also proposed by the recent findings that melatonin plays important roles in the protection of PSII by attenuating production of excess excitation energy and controlling ROS regulatory systems via the MITOGEN-ACTIVATED PROTEIN KINASEs (MAPKs) cascade involving MAPK3 and 6 [56,57].

HSPs are also known to be essential for the protection of PSII against heat stress [36,58]. Of these, HSP21 might play pivotal roles. It was revealed that HSP21 in *Arabidopsis* binds to a chloroplast nucleoid protein, PLASTID TRANSCRIPTIONALLY ACTIVE 5 (PTAC5), to maintain the chloroplast-encoded RNA polymerase [19]. HSP21 was also shown to directly bind to D1 and D2 proteins to protect PSII against heat stress as well as oxidative and photo-inhibitory stresses [59]. FILAMENTATION TEMPERATURE SENSITIVE H6 (FTSH6), a plastid-localized metalloprotease, functions as a negative regulator of HSP21 protein accumulation [56]. Deficiency in FTSH6 protein resulted in enhanced accumulation of HSP21 during heat stress, but an increase in FTSH6 protein oppositely affected accumulation of HSP21 during the recovery phase following heat treatment [60]. A more recent study demonstrated that HSFA2 acts as a regulator of both HSP21 and FTSH6 to modulate heat memory, an ability of plants to maintain high activity of heat response to better prepare for future heat stress even after the recovery from heat stress [61]. In tall fescue, FaHSP17.8-CII was shown to regulate heat tolerance of plants by modulating electron transport of PSII and ROS signaling [62]. The function of FaHSP17.8-CII is also associated with heat memory. Following the exposure to sublethal heat stress, heat memory of FaHSP17.8-CII was kept at high level for more than 4 days. During this heat memory phase, ROS accumulation increased and PSII electron transport was maintained [62]. In addition, heat stress causes the aggregation of LIGHT-HARVESTIG COMPLEX II (LHCII) proteins. During this LHCII aggregation, excess monogalacosyldiacylglycerol (MGDG), a main thylakoid membrane lipid associated with LHCII can be extruded [63]. Then, extruded MGDG forms inverted hexagonal phase (HP) [64,65], which is involved in the regulation of HSPs [66]. HP produced under heat stress activates VIOLAXANTHIN DEEPOXIDASE (VDE) involved in the xanthophyll cycle [67]. VDE synthesizes zeaxanthin that quenches excess excitation energy and stabilize the thylakoid membrane. These findings suggest that the integration of HSPs-dependent mechanisms with ROS regulatory systems is essential for the protection of photosynthetic reactions in the thylakoid against heat stress and oxidative stress.

Effects of heat stress on plant cells and acclimatory mechanisms to heat stress might be differently modulated depending on intensity of stress [68,69]. For example, in *Paspalum wettsteinii*, mild heat stress (40 °C or less) promoted growth, although severe heat stress (45 °C) resulted in excess accumulation of ROS accompanied by damages on photosynthetic machineries [69]. A recent study demonstrated that effects of heat stress on the chloroplasts in *Chlamydomonas reinhardtii* were different depending on the temperatures [68]. Moderate heat stress (35 °C) resulted in upregulation of the transcripts and proteins involved in carbon metabolisms as well as enhanced growth. In contrast, more severe heat stress (40 °C) resulted in defects in cell division and growth accompanied by disruption in thylakoid ultrastructure and decreased photosynthetic activity. During recovery following both mild and severe heat treatments, accumulation of the transcripts and proteins involved in DNA synthesis increased, although those involved in photosynthetic light reactions declined. Such effects on the mechanisms of DNA synthesis were more obviously observed following the exposure of plants to severe heat stress. Based on these findings, it was proposed that downregulation of photosynthetic light reactions during DNA replication might benefit recovery of the cell cycle by reducing ROS production [68].

Taken together, these findings suggest that damages on PSII leading to the inhibition of electron transport and ATP synthesis negatively impacts the activities of the CO_2_ fixation under heat stress. Thus, it is essential to protect functions of PSII against heat stress via the integration among multiple pathways involving HSPs and ROS regulatory systems. It should be interesting to investigate how these multiple pathways are flexibly coordinated depending on intensity of heat stress or growth stages. Effects of heat stress on the chloroplasts can also impact on functions of the mitochondria and energy homeostasis. Effects of heat stress on the mitochondria and the integrations between the chloroplasts and mitochondria will be discussed in the sections below.

## 3. Effects of Heat Stress on the Mitochondria and Mechanisms to Counteract Them

In contrast to photosynthesis that can be dramatically inhibited by heat stress, rate of respiration increases as temperature increases [4]. Like other biochemical processes, respiration rate increases about twofold for every 10 °C of the temperature increase. It occurs at highly variable temperatures of 40–60 °C, depending on the plant species and growth habitat [70,71]. Such an increase in respiration could be beneficial for cells to fulfill energy demand for the maintenance of cellular metabolisms under heat stress. However, due to the inhibition of photosynthesis, substrates (i.e., sugars produced by photosynthesis) for the metabolisms associated with respiration can be depleted and plants are required to strictly modulate energy usage. Thus, inhibition of respiration by heat stress can be also occurred because of the detrimental damages on the chloroplasts [17,25]. It has been also demonstrated that excess ROS production in seeds caused disruptions in mitochondrial functions under heat stress [72]. In addition, heat stress reduces not only the fixation of CO_2_ via suppression of photosynthesis but also activities of sucrose synthesizing enzymes [73]. In this case, the decline of the sugar supply required for respiration might be supported by the degradation of alternative sources such as starches and proteins to maintain ATP production [74,75,76].

Under the situations in which sugar supply is depleted, reduction of ATP demand might be essential to decrease energy costs during heat stress. Such an “energy-saving mode” can be achieved at least partially by decrease in photosynthetic rate, transport and synthesis of sucrose, and growth inhibition [4]. However, ATP costs for protein synthesis and maintenance can increase during heat stress because the stress causes denaturation of proteins. Probably, stabilization of proteins requires less energy compared to synthesis of proteins [4]. The HSPs-dependent maintenance of protein conformation could therefore contribute to stabilization of proteins under heat stress, resulting in a decrease of energy costs. However, this hypothesis is still controversial because HSPs also utilize ATP for their functions [7,28]. Another possible strategy to efficiently acclimate to heat stress with reducing energy costs is the formation of heat-stable isoforms of mitochondrial proteins. For example, mitochondrial MALATE DEHYDROGENASE adaptable to higher temperature with greater thermal stability was found in beach pea and *Arabidopsis* [4]. Thus, it is possible that reprograming of the proteome involving heat-stable isoforms of proteins might allow respiration to acclimate to higher temperature with lowered energy costs [4].

Increase in membrane fluidity caused by heat stress also affects respiratory energy demand of cells. Leakage of protons to the intermembrane space of the mitochondria inhibits ATP synthesis via excess consumption of substrates for the maintenance of the proton motive force required for ATP synthesis [77]. Decrease in the proton motive force for ATP synthesis results in inhibition of electron flow in the mitochondrial electron transport chain [78]. In addition, heat stress can inhibit the activity of CYTOCHROME C OXIDASE (COX) due to the membrane disruption. Inhibition of COX activity results in over-reduction of the ubiquinone pool in the electron transport chain, leading to transfer of excess electron to oxygen to produce excess ROS [79,80].

Mitochondrial damage caused by oxidative stress can trigger the release of metals from mitochondrial proteins [81]. The released metals directly affect activities of the electron transport chain in the mitochondria, leading to further ROS production [82,83]. Oxidation of target proteins is also catalyzed by the released metals [84,85], which triggers the mitochondrial dysfunction as well as excess ROS production [81]. Aggregation of proteins oxidized by the released metals is known to disturb mitochondrial functions [85]. The release of metals in the presence of high level of •O_2_^−^ and H_2_O_2_ accelerates the reaction to produce hydroxyl radical (•OH) that causes damages on the membrane via lipid peroxidation [81,86]. •OH can produce reactive carboxyl species (RCS) that lead to the formation of reactive compounds [87]. One of these RCS, 4-hydroxy-2-nonenal (HNE) was shown to inhibit several enzymes involved in the TCA cycle and respiration, such as MALATE DEHYDROGENASE, α-KETOGLUTARATE DEHYDROGENASE and PYRUVATE DEHYDROGENASE [88,89]. HNE also negatively impacts on the function of AOX. However, effects of HNE to the activity of AOX is still controversial because inhibition of respiration caused by HNE can also enhance accumulation of the *Aox* transcript and AOX protein [89].

Plants possess mechanisms to acclimate to heat stress via modulation of mitochondrial functions. Purified mitochondrial HSP22 from pea was shown to be decomposed into monomers in response to heat stress, preventing heat aggregation of rhodanese that detoxifies cyanide ion [90]. Roles of mitochondrial HSPs in the regulation of cellular ROS level during heat stress have been also reported. HSP24.7 in cotton was shown to positively regulate seed germination via temperature-dependent ROS generation [91]. In *Arabidopsis*, mitochondrial HSP70 is required to reduce ROS level and maintain COX activity during heat stress [92]. Several small HSPs were also reported to play important roles in protecting the electron transport chain and limiting ROS level during heat stress in apple and tomato [93,94,95]. Roles of sHSP24.1 in the response of eggplant to severe heat stress has been revealed in a recent study [21]. Overexpression of sHSP24.1 resulted in enhanced tolerance to heat stress up to 52 °C accompanied by upregulation of the transcripts involved in ROS scavenging, mitochondrial electron transport chain, auxin biosynthesis, and cell wall remodeling. These results suggest that the HSP-dependent pathways required for the protection of the mitochondria against oxidative stress play pivotal roles in the regulation of heat tolerance of plants, and at least to some extent, these pathways are conserved among various plant species. AOX has been well known to dissipate excess reducing equivalent to prevent over-reduction of the ubiquinone (UQ) pool without producing ROS [96,97]. Significance of AOX in the protection of plant cells against heat stress has been also reported. For example, increase in AOX capacity might contribute to acquired heat tolerance in spring wheat [26]. In marine diatom (*Phaeodactylum tricornutum*), downregulation of AOX resulted in enhanced sensitivity to heat stress accompanied by higher accumulation of H_2_O_2_ compared to WT [27], suggesting the role of AOX in the regulation of cellular ROS level under heat stress. In addition, AOX could be one of the key mediators of integration between the chloroplasts and mitochondria via retrograde signaling. These key roles of AOX will be discussed in the sections below.

In plant cells, proline is a multifunctional amino acid involved in responses to multiple stresses [98,99]. PROLINE DEHYDROGENASE (ProDH), a proline-degrading enzyme was shown to provide electrons to the electron transport chain in the mitochondria [100]. Under stress conditions accompanied by over-reduction of the electron transport chain, FADH_2_ in ProDH can reduce oxygen resulting in production of •O_2_^−^ and subsequently H_2_O_2_ [101]. The presence of proline and the activity of ProDH might be therefore associated with status of ROS production and signaling in the mitochondria [81]. It is still controversial how proline affects heat stress responses in plants. Toxicity of proline under heat stress was demonstrated in *Arabidopsis* [102]; however, positive roles of proline on heat tolerance of plants have been also reported (summarized in a book chapter [103]). Effects of proline on heat responses in plants could be different depending on the coordination with ROS level and status of other metabolisms that can be modulated depending on the intensity and duration of stresses.

A recent study demonstrated the involvement of the machineries regulating mitochondrial gene expression in heat response of plants. Suppression of TRANSCRIPTION TERMINATION FACTOR-RELATED 18/SUPPRESSOR of HOT1-4 1 (mTERF18/SHOT1) resulted in enhanced heat tolerance of *Arabidopsis* via attenuation of oxidative damage [104]. SHOT1 was found to bind DNA and localizes to the mitochondrial nucleoids. As SHOT1-interacting proteins, three homologues of animal ATPase family, AAA DOMAIN-CONTAINING PROTEIN 3 (ATAD3) were identified. Importantly, ATAD3 was shown to be involved in mitochondrial nucleoid organization, and deficiency in the function of ATAD3 resulted in disruption of the nucleoids, reduction in accumulation of the complex I, and enhancement of heat tolerance. Thus, such alterations caused by the deficiency in SHOT1 might activate the mechanisms to maintain mitochondrial metabolisms, leading to activation of heat acclimatory responses [104].

Taken together, the findings above suggest that heat stress can highly impact on membrane feature and activity of the electron transport chain in the mitochondria, which results in further generation of ROS leading to damage on the enzymes involved in respiration and related metabolisms. Thus, ROS regulatory systems and the mechanisms responsible for protections of the membrane and the electron transport chain against oxidative stress should be essential to regulate heat stress responses of plant cells. In future studies, it is necessary to elucidate how these heat acclimatory mechanisms in the mitochondria link to the systems to decrease energy costs. In addition, it should be also important to investigate how these heat acclimatory mechanisms are associated with other mechanisms involving proline metabolism and mitochondrial gene expression.

## 4. Heat Responses of Plants Regulated by Retrograde Signaling

**Figure 1:**
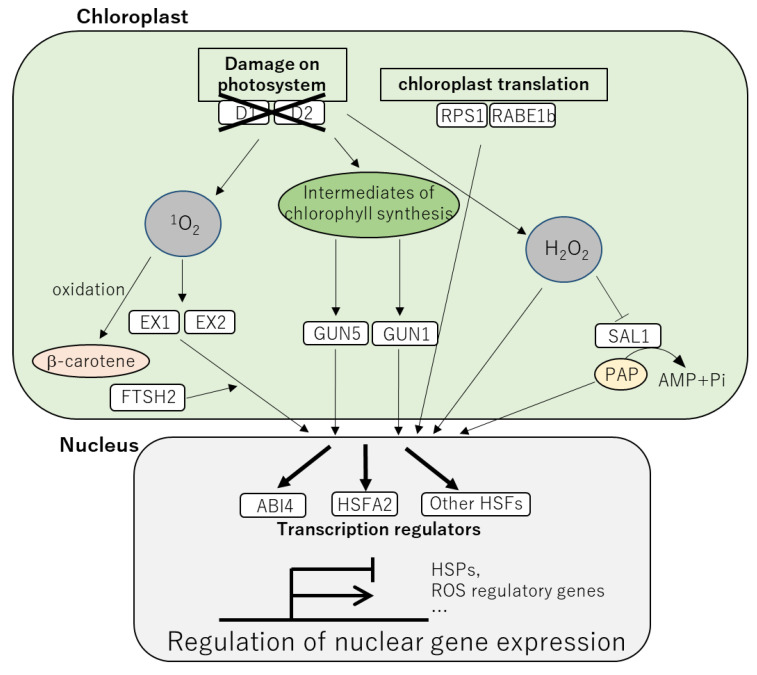
Retrograde signaling from the chloroplast associated with heat response in plants. ROS (^1^O_2_ and H_2_O_2_) and the intermediates of chlorophyll synthesis function as key regulators of retrograde signaling that activates or inhibits several proteins. These proteins activated via ROS or the chlorophyll intermediates transfer the signals from the chloroplasts to the nuclei, leading to activation or inhibition of nuclear gene expression.

Retrograde signaling from the chloroplasts and mitochondria to the nucleus optimizes functions of these organelles under stress conditions [105]. Previous studies demonstrated the involvement of chloroplast retrograde signaling in the regulation of heat response pathways (Figure 1). Retrograde signaling regulated by GENOME UNCOUPLED 5 (GUN5), the H-subunit of Mg-chelatase was shown to activate expression of the nuclear transcript encoding HSP21 that moves to the chloroplasts and protects D1 and D2 proteins against heat stress [59]. GUN1 activated by Mg-ProtoIX, a precursor of chlorophyll synthesis, functions upstream of ABA INSENSITIVE 4 (ABI4) that negatively or positively regulates transcription of downstream transcripts depending on binding targets [106,107]. Although detailed mechanisms of GUN1- and ABI4-dependent heat response in plants are still unknown, significance of these proteins in heat tolerance of plants was previously reported [108].

Protein translation in the chloroplasts is known as a heat-sensitive process [36]. Translation elongation factor Tu (EF-Tu), named RAB GTPASE HOMOLOG E1b (RABE1b), was shown to be important for the maintenance of translation in the chloroplasts under heat stress [109]. Downregulation of RABE1b in *Arabidopsis* and tomato resulted in enhanced sensitivity to heat stress as well as compromised expression of the transcript encoding HSFA2 [109]. Downregulation of RIBOSOMAL PROTEIN S1 (RPS1) in the chloroplast also resulted in impaired expression of the HSFA2-dependent heat response transcripts in the nucleus [110]. These results suggest that translation in the chloroplasts is required for chloroplast retrograde signaling which activates heat response pathway regulated by HSFA2. More recently, Trosch and coworkers demonstrated that heat stress led to a specific translational repression of chlorophyll a-containing core antenna proteins in PSI and PSII in *Chlamydomonas reinhardtii* [111]. Pattern of translocation of the ribosome nascent chain complexes to the thylakoid membranes was altered by heat stress, which is characterized by increased accumulation of the chloroplast SIGNAL RECOGNITION PARTICLE 54 (cpSRP54)-bound ribosomes [111]. cpSRPs are known to modulate chlorophyll antenna size to maximize photosynthetic productivity [112]. Thus, plastid translation might be modulated depending on light harvesting capacity of chlorophyll antenna that can be affected by heat stress.

^1^O_2_ generated by the damage of PSII in the chloroplast is also known as a key regulator of retrograde signaling [3,18]. The transcriptional responses mediated by ^1^O_2_ were first found by the analyses of the *Arabidopsis flu* mutant [113,114]. When *flu* mutant is moved from dark to light condition, the mutant accumulates high level of ^1^O_2_ produced by protochlorophyllide, a photosensitizing chlorophyll precursor [115]. The ^1^O_2_-dependent retrograde signaling requires EXECUTOR1 and 2 (EX1 and 2) proteins localized in the thylakoid membrane [114,115,116,117,118,119]. The plastid ATP-dependent zinc metalloprotease, FTSH2 is also involved in the signal transduction regulated by EX1 and EX2 [43,120]. FTSH2 was shown to be integrated with an unfolded protein response-like mechanism that regulates quality control of proteins including HSPs and ROS detoxifiers [43]. These results indicate the possible links between ^1^O_2_-dependent retrograde signaling and pathways involving HSPs and ROS regulatory systems. In contrast to H_2_O_2_, ^1^O_2_ might not be moved to the nucleus because of its short half-life. Thus, more stable second messengers activated by ^1^O_2_ might enhance downstream pathways [121]. β-carotene oxidized by ^1^O_2_ might be one candidate of the second messenger. It was demonstrated that β-carotene oxidized by ^1^O_2_ can re-program the expression of stress response transcripts [121]. H_2_O_2_ was shown to inhibit a key regulator of retrograde signaling, 3′(2′),5′-BISPHOSPHOSPHATE NUCLEOTIDASE (SAL1), leading to accumulation of 3′-phosphoadenosine 5′-phosphate (PAP). PAP transported from the chloroplast to the nucleus is known to regulate the expression of the nuclear genes involved in responses to high light and drought as well as programmed cell death [122,123]. However, roles of SAL1-dependent retrograde signaling in heat response have not been reported.

Due to high sensitivity to heat stress, the chloroplast is considered as a sensor of heat stress [124]. Ca^2+^ concentration in the stroma was reported to be increased in response to heat stress without altering Ca^2+^ concentration in the cytoplasm, suggesting that sensors of heat stress in the chloroplasts might be regulated by Ca^2+^-sensing proteins [124]. Ca^2+^ channels in the plasma membrane were proposed as heat sensors [125], and their functions are tightly linked with ROS signaling during heat stress [5,7]. It is therefore interesting to investigate how Ca^2+^-dependent heat sensing in the chloroplasts can be integrated with ROS regulatory systems involved in heat responses in plant cells. In addition, it should be also necessary to address how heat sensing mechanisms in the chloroplasts are linked to the regulation of retrograde signaling.

**Figure 2:**
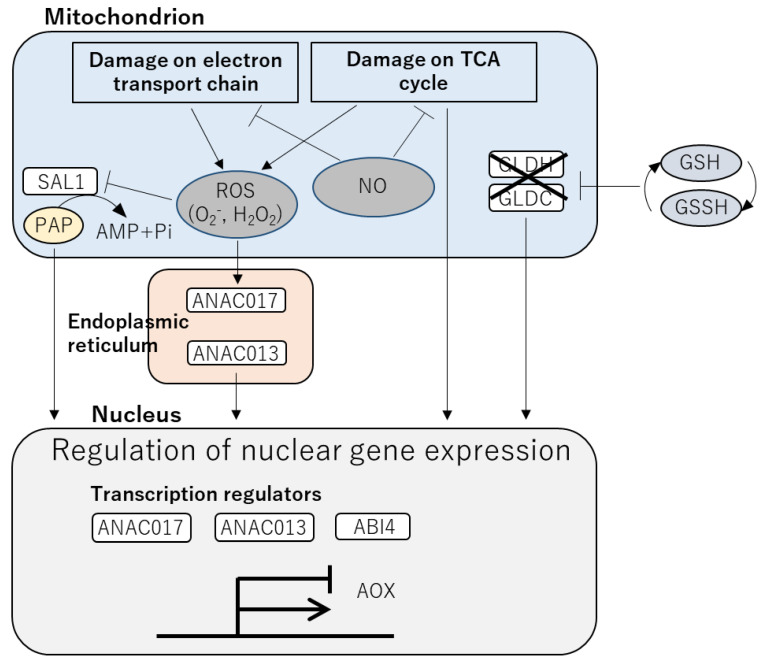
Retrograde signaling from the mitochondria that might be associated with heat response in plants. Damages on the electron transport chain or TCA cycle generate ROS. ROS can activate ANAC transcription factors that enhance expression of the transcript encoding AOX. In contrast, ABI4 activated by mitochondrial retrograde signaling inhibits AOX. Similar to the chloroplast, ROS can inhibit SAL1. Inhibition of mitochondrial enzymes (GLDH and GLDC) by S-glutathionylation might activate retrograde signaling. NO can also inhibit enzymes involved in the electron transport chain and TCA cycle via S-nitrosylation, leading to activation of retrograde signaling.

Several key players involved in mitochondrial retrograde signaling have been identified (Figure 2), although its underlying mechanisms are still largely unknown. AOX is a key marker to monitor the activation of mitochondrial retrograde signaling. ROS are known as signaling molecules that induce expression of the transcript encoding AOX (*Aox*). Previous studies demonstrated that exogenous application of antioxidants attenuated AOX activity induced by an inhibitor of the complex III of the electron transport chain [126,127]. Other regulators of mitochondrial retrograde signaling which modulate *Aox* expression were also identified by forward genetic screening in *Arabidopsis* [25]. Of these, ANAC017 was identified as a master regulator of mitochondrial retrograde signaling [128]. Although expression of the transcript encoding ANAC017 is not obviously altered by abiotic stresses, its protein conformational response to mitochondrial dysfunction has been revealed [128]. In response to perturbations of mitochondrial functions, the ANAC017 protein is released from the ER-membrane by the functions of an unknown protease and moves to the nuclei. ANAC017 was shown to function upstream to ANAC013, another key regulator of mitochondrial retrograde signaling [128].

Redox-dependent post translational modifications of proteins were also involved in the activation of mitochondrial retrograde signaling. Glutathione exists in the reduced (GSH) and oxidized (GSSH) forms and is known as a key regulator of cellular redox status [129]. S-glutathionylation is a reversible post translational modification that prevents cysteine residues of target proteins from oxidation [130,131]. Two mitochondrial enzymes, GALACTONOLACTONE DEHYDROGENASE (GLDH), a key enzyme in ascorbate synthesis, and GLYCINE DECARBOXYLASE (GLDC) involved in photorespiration, are known to be inactivated by S-glutathionylation [132,133]. The S-glutathionylation-dependent inactivation of these enzymes results in metabolic changes, which in turn triggers mitochondrial retrograde signaling [134]. S-nitrosylation by nitric oxide (NO) is also known as an important post translational modification of proteins involved in the regulation of mitochondrial retrograde signaling. Indeed, NO was shown to activate *Aox* expression [135,136]. Mitochondrial proteins targeted by S-nitrosylation include components of the complex I in the electron transport chain and ACONITASE, an enzyme that functions in the TCA cycle [136,137]. Inactivation of these proteins can induce increase in ROS as well as citrate. In addition, induction of *Aox* expression by monofluoroacetate (MFA), an inhibitor of ACONITASE in tobacco and Arabidopsis was reported in previous studies [138,139,140]. Application of MFA led to an increase in citrate level, and exogenous application of citrate also resulted in an induction of *Aox* expression in tobacco cell culture [139,141]. However, the role of citrate in the regulation of *Aox* expression is still unclear because *Aox* expression was not highly responsive to exogenous citrate application in Arabidopsis and soybean [142,143]. Probably, the significance of citrate in the regulation of retrograde signaling is a species-dependent process.

ABI4 was also identified as a mediator between mitochondrial retrograde signaling and expression of *Aox* transcript. ABI4 is known to be a repressor of *Aox* expression, which directly binds to the promoter of *Aox* gene [106,144]. In contrast, application of exogenous ABA can de-repress the inhibition of *Aox* expression by ABI4 [106]. Involvement of ABI4 both in chloroplast and mitochondrial retrograde signaling indicates the existence of the mechanisms that link these signals. Such integration of signals between the chloroplasts and mitochondria will be discussed in the following chapter.

Although AOX was shown to be involved in the protection of plants against heat stress [26,27], roles of its upstream regulators in heat responses are still largely unknown. It is necessary to address how disruptions of mitochondrial functions caused by heat stress can trigger retrograde signaling. In addition, it should be also important to investigate heat response transcripts and proteins that can be regulated by retrograde signaling.

## 5. Integration between Signals in the Chloroplasts and Mitochondria via ROS and Redox Regulation

**Figure 3:**
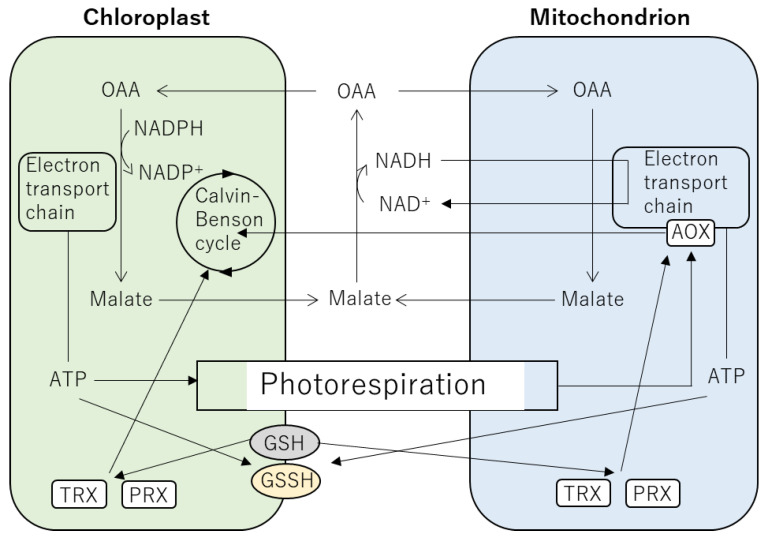
Integrations between the chloroplast and mitochondrion. Malate-OAA shuttle plays essential roles to prevent excess accumulation of reductants in the chloroplast, when photorespiration is accelerated. In this case, respiratory activity in the mitochondrion might shift to AOX-dependent to oxidize excess reductants in the mitochondrion. TRX and PRX modified by GSH in post-translation regulate proteins involved in the modulation of carbon assimilation, respiration and AOX.

To adapt to stress conditions including heat stress, it is necessary for plant cells to properly balance amounts of ATP, reductants and carbon via modulating functions of the chloroplasts and mitochondria (Figure 3) [145]. In the chloroplasts, conditions that accelerate the oxygenase activity of RUBISCO lead to high demand of ATP relative to NADPH [146,147,148]. In this case, a shortage of ATP relative to NADPH generation by the electron transport chain in the chloroplasts results in excess accumulation of stromal NADPH [145]. To shuttle excess reductants from the stroma of the chloroplasts, NADPH is consumed by the reduction of oxaloacetate to form malate. Then, malate transported from the chloroplasts to the cytosol is oxidized back to oxaloacetate (OAA) and returns to the chloroplasts [145]. This malate oxidation generates NADH, whose conversion to NAD^+^ might depend on the mitochondrial electron transport chain [145]. Thus, functions of the mitochondria to oxidize reductants excessively accumulated in the stroma of the chloroplasts might be essential to maintain redox status in cells. Such redox regulations might be important to maintain electron flow in the chloroplasts, leading to acceleration of the stromal ATP production. In addition, respiratory activity in the mitochondria might shift to AOX-dependent to oxidize excess reductants, when activity of photorespiration is enhanced. In Arabidopsis, the glycine to serine ratio increased under high temperature due to the enhanced photorespiration, and this trend was further accelerated in AOX1a-deficient plants [149]. These results suggest that mitochondrial GLYCINE DECARBOXYLASE (GDC), which converts glycine to serine during photorespiration, was inhibited by the deficiency in AOX1a at higher temperatures, probably by high matrix NADH [150,151]. It should be noted that photorespiratory activity of RUBISCO can be activated under high temperature [152]; thus, such redox regulations involving the chloroplasts and mitochondria should be essential for plants to adapt to heat stress.

Glutathione (GSH) is known to play important roles in the regulation of ROS and redox homeostasis as well as energy demand in the whole plant cell level [129,153,154]. Formation of disulfide bond between GSH and cysteine residues is an important post-translational modification of proteins [129,130] because it can prevent oxidation of protein thiols by ROS and alterations in protein conformation [130]. The redox regulatory proteins localized both in the chloroplasts and mitochondria such as thioredoxins (TRX) and peroxiredoxins (PRX) are the targets of the post-translational modification by GSH [155,156,157,158]. TRXs and PRXs have been also reported to regulate AOX and many target proteins involved in carbon assimilation and mitochondrial respiration [157,159]. Based on the localization both in the chloroplasts and mitochondria, it can be speculated that TRXs and PRXs might contribute to the cross talk between the mitochondria and chloroplasts [159]. Such cross talk between these energy-related organelles might be associated with the transport of metabolites including NAPDH, dihydroxyacetone phosphate, malate and glycolate, whose concentration can be altered depending on light and energy status. [159,160]. Ascorbate is present in the cytosol, chloroplasts and mitochondria and functions as a key antioxidant to detoxify ROS under stress conditions including heat stress [79,86]. Scavenging of H_2_O_2_ is depending on the functions of ASCORBATE PEROXIDASE (APX) that catalyzes oxidation of ascorbate to monodehydroascorbate. Reduction of monodehydroascorbate to ascorbate is carried out by the functions of MONODEHYDROASCORBATE REDUCTASES and GSH-DEPENDENT DEHYDROASCORBATE REDUCTASES [86]. Mitochondria are active sites producing ascorbate and utilizing ascorbate-glutathione cycle to scavenge H_2_O_2_, which can affect functions of the electron transport chain and oxidative phosphorylation [80,161,162]. Ascorbate produced in the mitochondria is provided to other cellular compartments including the chloroplasts. Thus, provision of ascorbate might affect chloroplast-derived signals that are depending on H_2_O_2_ [122,123,163].

Links between the chloroplasts and mitochondria can be also supported by the findings associated with retrograde signaling. The chloroplasts and mitochondria share the same components of retrograde signaling, such as ROS, ANAC017, ABI4 and PAP. Both chloroplasts and mitochondria produce •O_2_^−^ and H_2_O_2_. Of these, H_2_O_2_ with longer half-life compared to •O_2_^−^ moves into the cytosol and functions as a signaling molecule. It was demonstrated that in Arabidopsis, H_2_O_2_ interacts with the endoplasmic reticulum and induces release of transcription factors such as ANAC013 and ANAC017, key regulators of mitochondrial retrograde signaling [128,142]. Of these, ANAC017 was shown to be involved in the regulation of both chloroplast and mitochondrial retrograde signaling, which might suppress programmed cell death [164]. ABI4 and WRKY40 transcription factors were also known to regulate both chloroplast and mitochondrial retrograde signaling [81]. ABI4 acts as a repressor of the expression of the transcripts encoding LIGHT-HARVESTING CHLOROPHYLL a/b BINDING PROTEIN (LHCb) and AOX in the chloroplasts and mitochondria, respectively [105]. In contrast, WRKY40 and 63 positively regulate the expression of *Aox* transcript [105]. These WRKY transcription factors can be upregulated by dysfunctions of both chloroplasts and mitochondria, but not by a dysfunction of chloroplast or mitochondrial alone [165], suggesting their involvement in the modulation of the balance between functions of the chloroplasts and mitochondria. PAP, a common regulator of both chloroplast and mitochondrial retrograde signaling, might also contribute to the crosstalk between these organelles. Such a crosstalk can be supported by the fact that PAP- and ANAC017-dependent pathways shared by the chloroplasts and mitochondria are involved in the regulation of similar sets of transcripts [164]. In contrast to chloroplast retrograde signaling, detailed mechanisms of SAL1-dependent mitochondrial retrograde signaling are still largely unknown [81,105]. It is speculated that mitochondrial retrograde signaling might be modulated via the coordination between level of PAP accumulation, SAL1 activity and ROS homeostasis. However, export of PAP from the mitochondria to the cytosol has not been revealed [81]. To elucidate the crosstalk between chloroplast and mitochondrial retrograde signaling; therefore, it is necessary to further uncover the mechanisms underlying mitochondrial retrograde signaling.

## 6. Possible Involvement of Energy-Regulatory Hub in Heat Response of Plants

**Figure 4:**
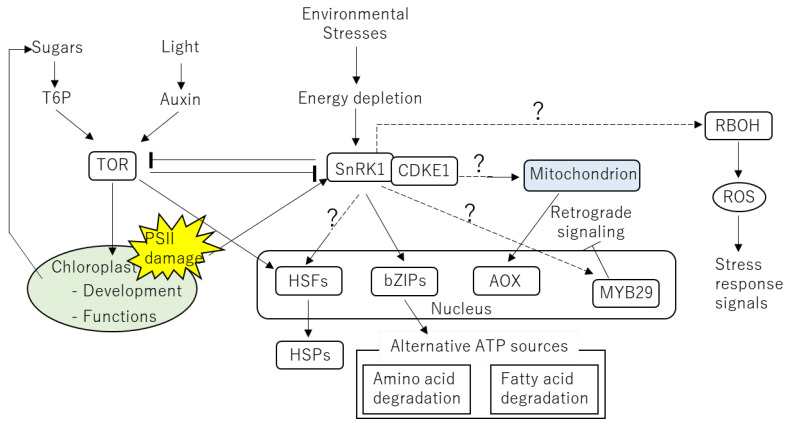
Putative links of TOR- and SnRK1-dependent energy regulation with functions of the chloroplasts and mitochondria during heat stress. TOR activated by the auxin and sugar signaling is required for proper activity of the chloroplasts. Glucose might also regulate expression of HSPs via TOR-dependent manor. In response to environmental stresses, energy depletion caused by the dysfunctions of the chloroplasts and mitochondria activates SnRK1. Then, SnRK1 activates stress responses involving AOX and bZIPs as well as mechanisms to obtain ATP from alternative sources. RBOH proteins required for the production of signaling ROS are also known to be activated by energy depletion, indicating the possible integration between SnRK1-dependent stress responses and RBOHs.

The functions of TOR and SnRK1 to modulate growth and stress response signals depending on energy status have been extensively studied [30,31,32]. However, majority of these research focused on the responses of plants to dark conditions or submergence. Unfortunately, it is still largely unknown how mechanisms involving TOR and SnRK1 function under heat stress. It can be however speculated that these mechanisms involving TOR and SnRK1 might also contribute to responses of plants to heat stress because functions of these proteins were shown to be linked to the heat-sensitive processes in the chloroplasts and mitochondria (Figure 4).

Glucose and sucrose derived from photosynthesis as well as light signals activate TOR signaling via promoting auxin signals, which enhance cell division in the shoot and root meristems [166,167,168,169]. Deficient plants in TOR showed severe defects in photosynthesis [18]. Overexpression of TOR also resulted in impaired chloroplast development [18]. These facts indicate that TOR might be involved in the maintenance of photosynthetic machineries. Under abiotic stresses responsible for energy deficit, in contrast to TOR accelerating energy consuming processes, SnRK1 restores the energy homeostasis by activating catabolic pathways to produce ATP, while inhibiting energy consuming pathways. Integration between functions of SnRK1 and regulation of photosynthesis has been demonstrated in several studies [170]. In *Arabidopsis*, AKIN10, a kinase subunit of SnRK1 was shown to be activated in response to the inhibition of electron transport at PSII [170]. Integration between ethylene signaling, photosynthesis and AKIN10 activity has been also reported in a previous study [171]. An *Arabidopsis* mutant deficient in ethylene response (*etr1* mutants) showed impaired PSII efficiency, leading to energy depletion that can activate AKIN10. Taken together, these results indicate that TOR and SnRK1 might control energy usage via modulating activity of photosynthesis depending on the energy status in cells.

SnRK1 can be activated by hypoxia accompanied by suppression of mitochondrial aerobic respiration and ATP synthesis [172,173]. Under such situations, BASIC LEUCINE ZIPPER 63 (bZIP63), a target of SnRK1 is known to form heterodimers with other bZIP family proteins. These bZIP transcription factors play important roles in amino acid breakdown under low energy conditions, which is known to be an alternative process of energy production. Under the situations with limited ATP production and low level of carbohydrates, retrograde signaling from the mitochondria can be also activated to induce alternative pathways of energy production such as catabolism of amino acids and fatty acids [174,175]. Amino acids can be also directly used as substrates to maintain the functions of the electron transport chain in the mitochondria under stresses accompanied by energy depletion [23]. Alterations in ATP level might affect level of other metabolites, leading to activation of mitochondrial retrograde signaling. These metabolites that act as retrograde signals include acetyl-CoA, the TCA cycle intermediates and NADH [174,176].

Involvement of SnRK1 in the regulation of AOX was demonstrated in previous studies. SnRK1 reprograms stress acclimatory mechanisms by modulating expression of the stress response genes and phosphorylation of the key metabolic proteins involved in mitochondrial retrograde signaling and the TCA cycle [81]. CYCLIN-DEPENDENT KINASE E1 (CDKE1) and MYELOBLASTOSIS DOMAIN PROTEIN 29 (MYB29) as well as several proteins involved in auxin signaling were identified as regulators of *Aox* expression [142,177,178]. CDKE1 was shown to interact with SnRK1 to activate AOX [142]. SnRK1-dependent activation of *Aox* expression is integrated with the primary energy/stress signaling pathways [34]. In contrast, inhibition of auxin signaling and MYB29 resulted in prolonged retrograde signaling, suggesting that they function as negative regulators of AOX. The role of auxin as a negative regulator of AOX can be also supported by research employing mutants deficient in auxin transporters [178].

Based on the links of TOR and SnRK1 with functions of the chloroplasts and mitochondria, we can speculate that TOR- and SnRK1-dependent mechanisms might be also associated with ROS regulatory systems. It has been well known that ROS producing enzymes, RBOHs are involved in a broad range of processes underlying growth, development and stress responses in plants [13]. Thus, the TOR- and SnRK1-dependent mechanisms could be integrated with the regulatory systems of ROS signals relying on RBOHs. Indeed, several RBOHs were shown to be upregulated in response to hypoxia accompanied by energy depletion [179]. Links between ROS regulatory systems and response to energy depletion can be also supported by the findings that a ROS specific *cis* element in the promoter of stress response genes functions under starvation [180,181]. In addition, a recent study demonstrated the significance of ROS in the modulation of the balance between cell division and differentiation in the shoot apical meristem [182], processes which can be regulated by TOR [166,167,168,169]. Abscisic acid (ABA) was shown to inhibit ATP-ADP exchange between the mitochondria and the cytosol by inhibiting mitochondrial ADENINE NUCLEOTIDE TRANSLOCATORs (ANTs) under the conditions accompanied by energy depletion [81,183,184]. Decrease in ADP in the mitochondria leads to accumulation of excess electrons in the electron transport chain and ROS production [81]. This inhibition of ATP-ADP exchange by ABA can be sensed by SnRK1, leading to subsequent reprograming of the expression of stress response transcripts and the phosphorylation of stress response proteins [185].

Previous studies indicated the possible links between TOR-dependent mechanisms with heat response pathways. Glucose-dependent TOR signaling was shown to induce expression of HSPs through the modulation of TOR-E2Fa signaling accompanied by enhanced heat tolerance [169,186]. Glucose also epigenetically regulates transcription of genes involved in heat responses in a TOR-dependent manner in which TOR functions together with p300/CREB HISTONE ACETYLTRANSFERASE 1 (HAC1). It has been demonstrated that HSP90 stabilizes auxin receptor TIR1 and promotes elongation of shoots and roots at warm temperature [187]. These results indicate the integration of the thermomorphogenesis involving auxin signals with the HSP-dependent pathway.

The findings above indicate that the TOR- and SnRK1-dependent mechanisms might play important roles in the regulation of heat responses in plants, because the processes in the chloroplasts and mitochondria which are linked with TOR- and SnRK1 are known to be heat-sensitive. In future studies, it is necessary to investigate how photosynthetic and respiratory processes damaged by heat stress can be modulated via functions of TOR and SnRK1.

## 7. Conclusions

Effects of heat stress on functions of the chloroplasts and mitochondria are mainly associated with excess production of ROS and altered redox status. ROS produced via dysfunctions of the chloroplasts and mitochondria can accelerate the damage on these organelles. However, increase in ROS production and alteration in redox status in these organelles can also trigger the acclimatory mechanisms of plants to heat stress. Indeed, many researchers have already known that ROS production needs to be strictly modulated under stresses including heat stress. The heat response mechanism activated by ROS include HSFs- and HSPs-dependent pathways. The findings described above suggest that plants possess various ROS-dependent pathways associated with the chloroplastic and mitochondrial signals to activate HSFs and HSPs in response to heat stress. Activation of heat responses via multiple pathways might be essential to flexibly tailor heat responses depending on intensity and duration of heat stress as well as growth stages of plants. We can expect that effects of heat stress on the chloroplasts and mitochondria might be different depending on such diverse situations with different energy demand.

Another key player regulating the heat acclimation of cells might be AOX in the mitochondria. Previous studies revealed various mechanisms that activate expression of *Aox* transcript. Even only for mitochondrial retrograde signaling, several key components responsible for the regulation of AOX expression have been found. In contrast to photosynthesis, to some extent, respiration can be activated by heat stress to provide energy required for protection of cells, indicating the significance of the maintenance of mitochondrial functions for the heat acclimation of plants. Thus, the modulation of ROS level via AOX might be a key process to protect cells against heat stress.

ROS and redox regulation might be essential to integrate functions of the chloroplasts and mitochondria via the exchange of metabolites and retrograde signaling, leading to the proper balance between ATP production and carbon level in cells. Significance of ROS in the regulation of carbon level and energy production could be also supported by the integrations of ROS regulatory systems with sugar metabolisms proposed in a previous review [188]. However, such integrations of mechanisms under heat stress still needs to be addressed in future studies. Despite the extensive studies focusing on effects heat stress on energy regulatory organelles (chloroplasts and mitochondria), roles of key energy regulatory mechanisms involving TOR and SnRK1 in heat response of plants are still not understood. The possible contributions of TOR and SnRK1 to heat responses of plants can be supported by the integration of TOR and SnRK1 with mechanisms involving HSPs, AOX and ROS production as well as functions of the chloroplasts and mitochondria. In future studies, it is necessary to uncover how TOR and SnRK1 regulate energy metabolisms to activate heat stress responses in plants.

## Data Availability

Not applicable.
